# Health economics and emergence from COVID-19 lockdown: the great big marginal analysis

**DOI:** 10.1017/S1744133120000304

**Published:** 2020-08-06

**Authors:** Cam Donaldson, Craig Mitton

**Affiliations:** 1 Yunus Centre for Social Business & Health, Glasgow Caledonian University, Cowcaddens Road, Glasgow G4 0BA, UK; 2 School of Population & Public Health, University of British Columbia, Vancouver, British Columbia, Canada

**Keywords:** COVID-19, lockdown, pandemic

## Abstract

Despite denials of politicians and other advisors, trade-offs have already been apparent in many policy decisions addressing the coronavirus disease 2019 pandemic and its social and economic consequences. Here, we illustrate why it is important, from a wellbeing perspective, to recognise such trade-offs, and provide a framework, based on the economic concept of ‘marginal analysis’, for doing so. We illustrate its potential through consideration of optimising the balance between reducing the reproductive rate (*R*) of the virus and further opening of the economy. The framework accommodates both perspectives in the health-vs-economy debate whereby, depending on where we are within the marginal analysis framework, either health issues are allowed to dominate or, below some threshold of *R* and/or background level of infection, health and economic considerations can be traded off against each other. Given the inevitability of such trade-offs, the framework exposes crucial questions to be addressed, such as: the critical value of *R* and/or background infection, above which health considerations predominate, and which may vary from jurisdiction to jurisdiction; and the value of lives forgone resulting from the small increases in *R* and/or background infection levels that may have to be tolerated as the economy is gradually opened.

## 1. Introduction

Trade-offs, trade-offs everywhere, but not a health economist in sight. It could be argued that this has been one of the puzzling things of the coronavirus disease 2019 (COVID-19) pandemic, given that resource scarcity and trade-offs are the very lifeblood of economics. Perhaps (health) economics is not ‘scientific’ enough. Perhaps laying bare the trade-offs being made would not be seen as helpful to politicians who, naturally, do not like to recognise that they exist. However, trade-offs have been palpable at all levels of decision making. The economic stimuli of governments across the globe have traded-off the future against the present and sectors of the economy with each other. Health and social care have been traded off against each other, with investments in large (often unused) health care capacity at the expense of services and equipment for people in care homes. Within health care, non-COVID-related care has been suspended to accommodate needs arising from the pandemic; initially for sound clinical reasons relating to do-no-harm, but less so now. From the very start, in many countries, shortages in testing and personal protective equipment were apparent, with meaningless large numbers thrown out in attempts to appease the public (e.g. the ‘100,000-per-day’ in the UK) and little recognition given to a more-systematic approach to what might be needed by different groups, from which priorities for access, based on health gain for resources expended, could be established.

Even economists would recognise that there is a level of pandemic where trade-offs do not matter, or, at least, are so obvious that little analysis is required. This is also because, beyond a particular level of *R* (the reproduction rate for the virus) and background prevalence and incidence, the economy and health considerations go hand-in-hand. Particularly, at certain levels of disease, *R* must be controlled. Also, economists can contribute to the more ‘science-like’ modelling work of various international research groups; and, likely, have been doing so. But surely, health economics has more to offer as a way of thinking and, thus, in contributing to honest debate about emerging trade-offs once *R* is controlled and the curve flattened. The trade-offs emerging now are those around lockdown. No more so than in the US, where Federal and State governments have often been at loggerheads over the health and economic trade-offs of releasing (and imposing) lockdown, as well as in the now-numerous other locations, internationally, where local lockdowns have been imposed. With public health leaders having done their best to flatten the curve in many jurisdictions and cases increasing in others, we are now in a new phase where a broader set of questions, and disciplines like health economics, are particularly relevant. This requires a framework which brings considerations of health and the economy together and to which (economic) modelling can contribute. It is necessary because, as indicated, the trade-offs are happening now, but without systematic analysis or public debate. Health economics can facilitate this using a theoretical and well-established framework known as ‘marginal analysis’.

## 2. Marginal analysis

An economic approach to priority setting simply has to adhere to two key economic concepts; ‘opportunity cost’ and ‘the margin’. Opportunity cost refers to having to make choices within the constraint of limited resources; certain opportunities will be taken up while others must be forgone. The benefits associated with forgone opportunities are opportunity costs (Karlsberg Schaffer *et al*., 2016). Thus, when applying such a framework within publicly funded health and social care, we would normally state that a consequence of opportunity cost, and in order to spend a limited budget to maximum effect, we need to know the costs and benefits from various health and care activities (Donaldson *et al*., 2002). Marginal analysis refers to the fact that assessment of costs and benefits is best addressed ‘at the margin’. The focus is on the benefit gained from the next unit of resources, or that lost from having one unit less. Imagine that resources are so scarce that we have to choose between elective heart and hip surgery programmes – perhaps not such an unrealistic prospect, even in parts of the US and in other well-funded health care systems, going forward in the short-to-medium term. The question is not one of whether to resource one whole programme and none of the other. Rather, it is one of balance between the two.

If marginal benefit (MB) per $/£ spent from, say, an elective heart operation programme is greater than that for an elective hip replacement, then, all else equal, resources should be taken from hips and given to hearts (Olsen and Donaldson, 1998). On the basic economic principle of ‘diminishing marginal benefit’, whereby physicians prioritise those with most to gain, MBs from hearts will begin to fall as the programme is expanded and those for hips rise, as less-beneficial care is withdrawn, until the two come into alignment, all else equal. Such reallocation would stop, therefore, at a point of balance, where the MB (relative to marginal cost (MC)) for hearts and hips are equalised. Based on such thinking, marginal analysis frameworks have been developed and applied in several hundred health organisations internationally (Mitton and Donaldson, 2004; Peacock *et al*., 2006; Tsourapas and Frew, 2011). Indeed, we would also contend that such frameworks, and the way of thinking they embody, could have been used in earlier stages of the pandemic and even in preparation for it. Unsurprisingly, a ‘great big marginal analysis’, of the sort currently required and which we now outline, has never, to our knowledge, been undertaken.

## 3. The great big marginal analysis: emerging from lockdown

**Figure 1.**
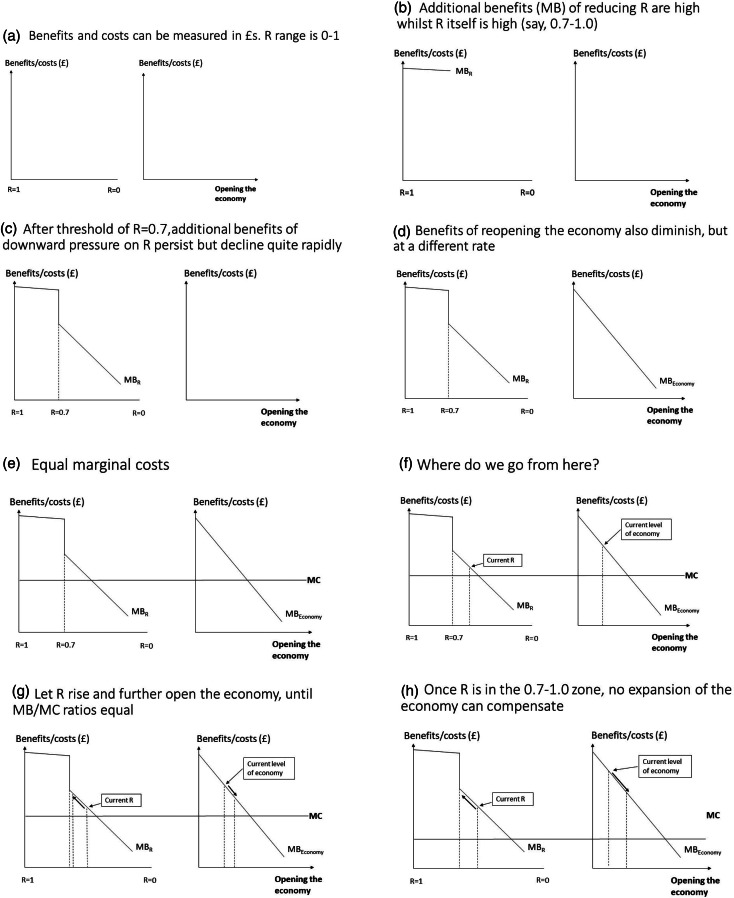
Great big marginal analysis: the theory.

Often, the thought process behind marginal analysis is better described via a sequence of conceptual diagrams, which we now do in the context of emerging from lockdown – see Figure 1. The assumptions, to illustrate, are that we are now in a zone where the background level of infection is still significant but in which *R* is less than 1. We are, of course, aware that *R* differs by subgroups, across time and by location, but this does not deflect from the substance of the argument that trade-offs with the economy, whether at local, regional or national levels, can be made; and, indeed, *are* being made. This has often been denied by politicians in many countries, but is now, further into the pandemic and the associated adverse consequences of lockdown, more-openly discussed. The ‘benefits’ being traded-off refer to some abstract notion of aggregate community well-being. Such benefits can arise from individual and private sector activity, but, in the current context, is heavily overlain and intertwined with public ‘bads’ and negative externalities (in the form of communicable disease), and, conversely, with improvements delivered by public goods and positive externalities (e.g. future protection). Therefore, given that only collective (i.e. not individual) actions can deliver such public goods and internalise such externalities, the framework is one proposed for use and adaption by government and other public agencies.

For purposes of illustration, we are saying that such community benefit can be monetised and so presented on the same scale (or axis) as costs (Figure 1a). Diagrams to the left in Figure 1 illustrate MCs and benefits of reducing *R* from 1 to 0 whilst those to the right illustrate the same for opening the economy. In the latter, we include all sectors; public, private and civil society.

First, we invoke the above-mentioned notion of diminishing MB, whereby the MBs of reducing the *R*-value from 1 is positive but gradually reduces. Let us say that in the range of *R* = 0.7–1.0, MB reduces only very gradually (1b), after which, due to it being less critical, a sudden drop off in MB occurs before it continues on a more-regular downward slope (1c). Accordingly, and to be clear, as we reduce *R*, the total benefits are greater and greater, but the increases, in terms of the marginal social value of the corresponding health gains, are lesser and lesser. Hence, the downward sloping MB line for *R* and, correspondingly, for ‘Opening the economy’ (1c and 1d). Of course, we would recognise that, in the latter case, people may debate which sectors, or even which parts of sectors, are more or less valuable, even at the margin. However, even within sectors, we assume that the most-needed parts will be opened first, or, of course, never close. To aid presentation, assume the MC of reducing *R* or economic expansion are constant and equal (1e). The question then becomes, given, on any particular day, a particular starting point for each of *R* and the economy, where do we go from here (1f). From 1 g, it can be seen that the gap between MB and MC (or the MB/MC ratio) at the chosen starting point for the economy is greater than at the starting point for *R*. Thus, the economy can expand and *R* can be allowed to drift upwards as we trade-off the gains from the former against the losses (which there will inevitably be) from the latter. Opening of the economy can continue until the MB/MC ratios are equalised. The final diagram (1 h) shows that, when a consequence of any economic expansion is for *R* to fall back into 0.7–1.0 range, the marginal gains from any such expansion will be too small to justify going past a certain point, beyond which the gains from focussing on *R* far outweigh those of the economy.

## 4. Conclusion

It is important to reiterate that we are not claiming that the lines in Figure 1 can be measured accurately. They are used to represent an important debate currently ensuing throughout the world as well as a thought process to aid its resolution. It is a thought process which accounts for the two main perspectives in ongoing ‘health-vs-the-economy’ debates. Depending on where we are within the marginal analysis framework, either health issues dominate, only because they go hand-in-hand with the economy, or, below some threshold of *R* and/or background level of infection, health and economic considerations can be, and we would contend are, traded off against each other.

Moving forward with such a framework has two main implications. The first is that criteria are required to reflect what we mean by ‘benefit’ (Peacock *et al*., 2007). In the current context, this must be done with respect to reducing *R*, extent of infection and opening of economies. Many governments have defined sets of criteria for each, but have not connected them in one framework. Second, and relatedly, nor have many governments openly admitted the need for trade-offs, not only within which lives may be lost but also tolerated. Indeed, it might be argued that deliberately not recognising trade-offs then allows avoidance of such uncomfortable truths. Even when below some critical threshold (like 0.7 in our example), a small increase in *R* will result in more infections and, as just alluded to, risk to life. Note current international debates on ‘1 m vs 2 m’ on social distancing. Is there an amount of economic expansion which might provide benefits to the population so great by which an upward drift in *R*, with its consequent risks, would be permitted?

Such issues require debate and evidence on at least two key things: the critical range within which concern for *R* and background infection levels take over any concern even for incremental opening of the economy, a range which may, of course, vary across jurisdictions; and more explicit use of values of health and life which already exist and are used in public policy (Viscusi and Aldy, 2003; Donaldson *et al*., 2011; Baker *et al*., 2014). The latter are based on asking representative samples of the population about the value they place on small changes in risk (Chilton *et al*., 2020). Their use, ultimately, means facing up to the question of the value to be placed on life and health vs opening the economy *at the margin*, accepting that that value may change according to whether we are above or below a critical value of *R*. In our view, the trade-offs referred to in this paper are inevitable and, for purposes of optimising overall human welfare, are better recognised, analysed and publicly debated.
